# Seminal Plasma and Extracellular Vesicles as Molecular Gatekeepers: Oxidative Stress, Endocrine Crosstalk, and Biomarker Discovery in Male Infertility

**DOI:** 10.3390/cimb48010117

**Published:** 2026-01-22

**Authors:** Pallav Sengupta, Sulagna Dutta, Mahir Khalil Jallo, Israel Maldonado Rosas, Shubhadeep Roychoudhury

**Affiliations:** 1 Department of Biomedical Sciences, College of Medicine, Gulf Medical University, Ajman 4184, United Arab Emirates; 2 Basic Medical Sciences Department, College of Medicine, Ajman University, Ajman 3464, United Arab Emirates; 3 Centre of Medical and Bio-Allied Health Sciences Research, Ajman University, Ajman 3464, United Arab Emirates; 4 Center of Internal Medicine & Endocrinology, Thumbay University Hospital, Ajman 4184, United Arab Emirates; 5 Citmer Reproductive Medicine, Mexico City 11520, Mexico; 6 Department of Life Science and Bioinformatics, Assam University, Silchar 788011, India

**Keywords:** reproductive outcomes, metabolic regulators, endocrine signaling, reactive oxygen species, semen

## Abstract

Conventional semen analysis fails to capture the molecular determinants underlying impaired reproductive function. Emerging evidence positions seminal plasma (SP) and extracellular vesicles (EVs) as dynamic regulators of sperm physiology, rather than passive transport components. SP, enriched with proteins, metabolites, hormones, and antioxidants, modulates sperm motility, capacitation, acrosome reaction, and immune tolerance. Complementarily, EVs, including prostasomes, epididymosomes, and testicular vesicles, deliver proteins, lipids, and small RNAs that remodel sperm membranes, protect against oxidative insults, and influence fertilization success. A critical dimension of the SP-EV axis is its role in balancing oxidative stress (OS) and endocrine signaling. Hormones and metabolic regulators within SP, together with EV-mediated transfer of receptors and regulatory RNAs, further integrate systemic metabolic health with local reproductive outcomes. Dysregulation of these networks, particularly in conditions such as varicocele, obesity, diabetes, and idiopathic infertility, compromises sperm function and reduces assisted reproductive technology (ART) success. This evidence-based review synthesizes current evidence on SP and EVs as ‘molecular gatekeepers’ in male infertility, emphasizing OS regulation, endocrine crosstalk, and their potential as biomarker reservoirs. By integrating proteomic, metabolomic, and transcriptomic insights, the translational opportunities for biomarker-informed diagnostics, prognostication, and therapeutic interventions are highlighted.

## 1. Introduction

Male infertility constitutes a major public health concern, contributing to nearly half of all infertility cases worldwide [1]. Current estimates indicate that around 7–12% of men of reproductive age are affected by infertility issues, with significant psychosocial and economic implications for couples and healthcare systems [2]. While conventional semen analysis provides essential information, it fails to capture the intricate molecular determinants that govern sperm development, maturation, and fertilization [3]. Increasing evidence highlights that infertility is not merely a disorder of gamete number or motility but a complex dysfunction of cellular and molecular processes within the male reproductive tract [4].

Among these, seminal plasma (SP) and extracellular vesicles (EVs) have emerged as critical mediators. Traditionally considered as passive carriers or supportive fluids, SP and EVs are now recognized as dynamic regulators of sperm function [5]. SP, secreted from accessory glands, is enriched with proteins, metabolites, hormones, and antioxidants that modulate motility, capacitation, acrosome reaction, and immune tolerance [6]. Similarly, EVs, including prostasomes, epididymosomes, and testicular vesicles, deliver bioactive cargo such as proteins, small RNAs, and lipids that directly influence sperm membrane remodeling, fertilization competence, and protection from oxidative insults [7]. Together, the SP-EV axis orchestrates a fine molecular dialogue essential for reproductive success [5].

Despite these advances, perturbations in redox homeostasis and endocrine signaling represent pivotal threats to sperm integrity [8,9]. Oxidative stress (OS), often originating from defective mitochondria or infiltrating leukocytes, drives DNA fragmentation, lipid peroxidation, and protein oxidation [10]. Concurrently, altered endocrine and metabolic signals, such as insulin resistance, obesity, and hormonal imbalance, translate into impaired SP-EV composition and dysregulated sperm responses. This interplay suggests that oxidative imbalance and endocrine crosstalk converge at the SP-EV interface, thereby shaping male fertility outcomes.

This evidence-based narrative review aims to critically synthesize emerging evidence on SP and EVs as ‘molecular gatekeepers’ in male infertility. Specifically, we highlight their role in OS regulation, endocrine communication, and their promise as biomarker reservoirs. By integrating these dimensions, the review seeks to bridge molecular insights with translational prospects in diagnosis, prognosis, and therapeutic interventions for male infertility.

This narrative review was conducted in accordance with the Scale for the Assessment of Narrative Review Articles (SANRA). The literature was identified through targeted searches of major scientific databases, including PubMed, Scopus, and Web of Science, using relevant keywords related to seminal plasma, extracellular vesicles, oxidative stress, endocrine signaling, and male infertility. Peer-reviewed original research articles and authoritative reviews published in English were prioritized based on relevance, scientific quality, and contribution to the thematic focus of this review. The selected literature was critically evaluated and synthesized to ensure balanced coverage, logical structure, and integration of mechanistic, clinical, and translational perspectives, in line with SANRA recommendations.

## 2. Seminal Plasma: More than a Transport Medium

### 2.1. Composition of Seminal Plasma

SP, which constitutes over 90% of the ejaculate volume, represents a highly complex and dynamic fluid enriched with biomolecules secreted from the prostate, seminal vesicles, epididymis, and bulbourethral glands [11]. Its composition is not merely a byproduct of glandular secretions but reflects a carefully orchestrated microenvironment that supports sperm survival, function, and transport [12]. Proteomic analyses have identified thousands of proteins in SP, including enzymes, structural proteins, cytokines, and signaling mediators, many of which are directly involved in sperm protection and fertilization competence [13]. Metabolites such as polyamines, citrate, fructose, and prostaglandins provide essential energy substrates and modulate the biochemical milieu critical for capacitation [14]. In addition to proteins and metabolites, SP is rich in inorganic ions (Ca^2+^, Zn^2+^, Mg^2+^, Na^+^, K^+^), which are central to motility regulation, acrosome reaction, and chromatin stabilization [15]. Hormones such as testosterone, estradiol, and cortisol are detectable in measurable concentrations and act synergistically with other local mediators to shape the reproductive tract’s functional state [16]. Importantly, SP harbors an array of enzymatic and non-enzymatic antioxidants–superoxide dismutase (SOD), catalase (CAT), glutathione peroxidase (GPx), uric acid, and vitamins C and E, that counterbalance reactive oxygen species (ROS) generated during sperm storage and ejaculation [17].

However, the composition of SP is highly variable across individuals and is significantly influenced by systemic health, age, diet, and underlying metabolic conditions [18]. For instance, obesity, diabetes, and varicocele alter the levels of key proteins and antioxidants, tipping the balance toward OS and impaired sperm physiology [19]. This compositional plasticity highlights SP’s dual role as both a protective medium and a potential reservoir of molecular signals reflecting systemic dysfunction. A critical appraisal of SP composition, therefore, provides a window into both male reproductive health and broader metabolic-endocrine status.

### 2.2. Molecular Roles in Sperm Function

Beyond its structural and nutritive role, SP is increasingly recognized as a central modulator of sperm physiology through molecular mechanisms. One of its primary functions is the regulation of sperm motility, achieved by providing substrates for mitochondrial energy production, modulating ion fluxes, and stabilizing the sperm plasma membrane [20]. Components such as fructose and citrate fuel ATP synthesis, while ions like calcium and zinc critically influence flagellar motion and acrosomal dynamics [21]. SP also plays an indispensable role in capacitation, the biochemical reprogramming of sperm required for fertilization. Capacitation is tightly regulated by SP proteins, glycoproteins, and lipids that modulate cholesterol efflux, membrane fluidity, and cyclic AMP signaling. Glycosaminoglycans, for example, act as capacitation modulators by influencing tyrosine phosphorylation cascades. Furthermore, SP regulates the acrosome reaction by fine-tuning the release of acrosomal enzymes essential for zona pellucida penetration [11,22].

The immunomodulatory role of SP extends beyond the male reproductive tract and critically depends on factors that shape fertility outcomes in females. Rich in immunoregulatory cytokines, transforming growth factor-β, prostaglandins, and EV-associated molecules, SP promotes a controlled immune response within the female reproductive tract that supports sperm survival while limiting immune rejection. Upon insemination, SP and EVs modulate endometrial immune activity by regulating leukocyte recruitment, macrophage function, and cytokine signaling, thereby contributing to endometrial receptivity and maternal tolerance to paternal antigens. EV-mediated delivery of complement inhibitors and immune-regulatory factors further protect sperm from immune-mediated damage. Disruption of these female immune and endometrial responses can impair the beneficial effects of SP and EVs, highlighting the importance of coordinated male–female immune interactions in successful fertilization and implantation [23]. Simultaneously, SP antioxidants form the first line of defense against oxidative and nitrosative stress, mitigating DNA fragmentation and lipid peroxidation that compromise fertilization potential [17]. However, the beneficial functions of SP can be compromised under pathological states. Elevated ROS levels or altered cytokine profiles transform SP from a protective milieu to a detrimental one, exacerbating oxidative damage, impairing capacitation, and reducing sperm viability [24]. Thus, SP serves as a double-edged sword, where its molecular constituents determine the balance between reproductive competence and infertility. Critically, deciphering these mechanisms provides insights into the pathogenesis of idiopathic infertility and guides the development of therapeutic interventions aimed at restoring molecular homeostasis within the ejaculate.

### 2.3. Endocrine and Paracrine Signaling Within Seminal Plasma

SP is not only a nutrient-rich suspension medium but also a reservoir of hormones and metabolic regulators that mediate intricate endocrine and paracrine interactions [6]. Testosterone and estradiol, derived primarily from testicular and accessory gland secretions, are consistently present in SP, modulating sperm membrane stability, capacitation, and acrosome responsiveness [25]. Inhibin B, secreted by Sertoli cells, serves as a functional correlate of spermatogenesis, while cortisol, leptin, insulin, and adiponectin mirror the systemic metabolic milieu, linking reproductive outcomes to broader endocrine health [26].

These hormones act in concert with paracrine mediators such as growth factors, cytokines, and prostaglandins, which shape the local microenvironment within the male and female reproductive tracts. For instance, leptin and insulin within SP influence sperm metabolism and motility, whereas cortisol modulates stress responses that affect sperm survival [27]. Crosstalk between SP endocrine mediators and systemic metabolic stress has gained increasing attention. Men with obesity, metabolic syndrome, or diabetes exhibit altered SP hormone levels and signaling patterns, often associated with decreased sperm quality and reduced assisted reproductive technology (ART) success rates [28]. Notably, the metabolic-reproductive axis is further complicated by partner-related conditions such as polycystic ovary syndrome (PCOS), where altered hormonal landscapes may synergize with male SP dysfunction to compromise fertilization outcomes [29]. Paracrine signaling within SP also ensures communication between spermatozoa and the accessory gland environment. Prostaglandins and cytokines modulate immune responses and regulate oxidative balance, while vesicle-bound mediators facilitate localized delivery of bioactive molecules [30]. Dysregulation of these signaling networks, through chronic metabolic stress, inflammation, or endocrine disruption, can impair sperm function and compromise fertilization capacity [31]. Critically, understanding SP as an endocrine-paracrine signaling hub highlights its role as both a reflection and mediator of systemic health. This duality underscores the necessity of integrating reproductive endocrinology with metabolic medicine to fully elucidate the molecular determinants of male infertility.

## 3. Extracellular Vesicles in Semen: Messengers of Reproductive Communication

### 3.1. Types and Origins of Extracellular Vesicles in Semen

EVs are nano-sized, lipid bilayer-enclosed particles secreted by virtually all cell types and represent key mediators of intercellular communication [32]. In semen, EVs constitute a heterogeneous population derived from the male reproductive tract, reflecting the contributions of the prostate, epididymis, testis, and other accessory glands [33]. Based on their cellular origins and biochemical properties, three main EV subpopulations are recognized: prostasomes, epididymosomes, and testicular EVs, each carrying distinct molecular cargo and fulfilling specialized reproductive roles [7].

Prostasomes, secreted by the prostate gland, represent one of the best-characterized EV populations in semen [34]. They are typically 30–500 nm in diameter and enriched with cholesterol, sphingomyelin, tetraspanins, and functional proteins [35]. Prostasomes are heavily implicated in sperm motility regulation, membrane stabilization, and immunomodulation within the female genital tract. Their protein cargo includes enzymes (e.g., dipeptidyl peptidase IV, prostate-specific antigen), transporters, and antioxidant enzymes that protect sperm from OS. Moreover, prostasomes contain microRNAs and other small non-coding RNAs that can modulate sperm gene expression post-transcriptionally [36].

Epididymosomes, originating from epididymal epithelial cells, represent a critical EV class involved in sperm maturation [37]. During epididymal transit, sperm acquire proteins, lipids, and RNAs from epididymosomes through vesicle-sperm fusion or endocytic uptake [38]. These vesicles facilitate key steps in plasma membrane remodeling, acquisition of motility, and preparation for capacitation. Epididymosomes are also known to deliver chaperones and enzymes that regulate redox balance and stabilize sperm chromatin integrity. Importantly, their miRNA cargo contributes to epigenetic programming, with potential implications for paternal transmission of acquired traits [39].

Testicular EVs, although less extensively studied, emerge from Sertoli, Leydig, and germ cells within the seminiferous tubules. These vesicles participate in intra-testicular communication, influencing spermatogenesis and the survival of germ cells [40]. Recent evidence indicates that testicular EVs contain factors regulating meiotic progression, stress-response proteins, and RNAs implicated in spermatogonial stem cell maintenance [41]. Together, these EV populations create a multilayered communication network within semen, ensuring the functional maturation and protection of spermatozoa. Notably, their composition is highly dynamic, influenced by age, systemic metabolic disorders, environmental exposures, and reproductive pathologies such as varicocele or infection [42]. For instance, altered prostasome lipid composition and reduced antioxidant capacity have been associated with idiopathic male infertility. Similarly, epididymosomal miRNA profiles differ markedly between fertile and infertile men, positioning them as candidate biomarkers [43].

A critical challenge remains the lack of standardized methods for EV isolation and characterization in semen, leading to inconsistencies across studies. Ultracentrifugation, size-exclusion chromatography, and immunocapture yield vesicle populations of varying purity and functional relevance [44]. Addressing this methodological heterogeneity is crucial for advancing translational applications. In summary, semen-derived EVs constitute a diverse and functionally specialized group of vesicles that orchestrate molecular signaling in reproduction. Their origins and cargo not only regulate sperm competence but also provide a molecular reflection of male reproductive health [45].

### 3.2. Functional Role in Sperm Maturation and Fertilization

The functional importance of semen-derived EVs lies in their ability to modulate the sequential events that transform immature spermatozoa into fertilization-competent gametes [5]. These functions encompass sperm maturation, capacitation, acrosome reaction, and direct participation in sperm-oocyte interactions [22].

During epididymal maturation, spermatozoa, initially transcriptionally silent and functionally immature, acquire motility and fertilizing capacity through molecular cargo delivered by epididymosomes [39]. Proteins involved in membrane trafficking, lipid metabolism, and ion transport are transferred to sperm, facilitating the remodeling of the plasma membrane. Specific chaperone proteins, such as clusterin and heat shock protein 70 (HSP70), ensure proper folding and stabilization of newly delivered proteins, protecting sperm from oxidative insults [46]. Furthermore, epididymosomal small RNAs (notably microRNAs–miRNAs, and piwi-interacting RNAs–piRNAs) reshape the sperm epigenome, fine-tuning post-fertilization gene regulation and potentially influencing embryonic development [47]. Capacitation, a prerequisite for fertilization, is tightly regulated by EVs. Prostasomes modulate sperm cholesterol efflux, a central step in capacitation, by delivering cholesterol and sphingomyelin-rich membranes that buffer premature capacitation in the male tract and orchestrate the timing of capacitation within the female tract [48]. EV-derived enzymes also regulate cyclic AMP (cAMP) signaling and tyrosine phosphorylation cascades critical for capacitation [49].

The acrosome reaction, an exocytotic event enabling zona pellucida penetration, is influenced by EV cargo, including calcium-binding proteins, phospholipases, and fusogenic lipids [50]. Prostasomes enhance calcium influx in spermatozoa, priming them for acrosomal responsiveness [51]. Moreover, EVs contribute membrane lipids that facilitate the fusion of the sperm plasma membrane with the outer acrosomal membrane [5]. In terms of sperm-oocyte interaction, EVs exert multiple roles. Prostasomes carry adhesion molecules, integrins, and ligands that facilitate sperm-zona pellucida binding [52]. Certain EV-derived RNAs and proteins are internalized by oocytes upon sperm-oocyte fusion, potentially contributing to zygotic development. Notably, evidence suggests that paternal EV cargo may influence early embryonic gene expression, linking paternal environment and reproductive success [45,52].

The protective role of EVs against OS also critically underpins their functional contribution [53]. By delivering antioxidant enzymes (SOD, CAT, peroxiredoxins) and stress-response molecules, EVs prevent premature sperm senescence and preserve fertilization capacity [53]. This protection is particularly relevant in pathological conditions such as varicocele, chronic inflammation, or advanced paternal age, where oxidative insults are exacerbated [54]. Importantly, dysfunction of EV-mediated processes correlates with clinical infertility. Altered epididymosomal RNA cargo, impaired prostasome secretion, or reduced EV antioxidant capacity compromise sperm function, contributing to idiopathic infertility cases where conventional semen parameters remain normal [55]. These insights underscore the need to move beyond descriptive semen analysis toward the molecular evaluation of EV function. Thus, semen EVs represent indispensable effectors of sperm functional maturation and fertilization competence [55]. They ensure the precise timing of capacitation and acrosome reaction, maintain sperm viability, and contribute molecular signals to the fertilization process. Disruption of these EV-mediated functions directly compromises reproductive outcomes, positioning them as both biomarkers and therapeutic targets in male infertility.

### 3.3. Crosstalk with the Seminal Plasma Milieu

EVs do not act in isolation within semen but operate in dynamic interplay with the SP, creating a highly coordinated molecular ecosystem [5,33]. The SP milieu, enriched in proteins, metabolites, hormones, and antioxidants, provides both a medium for EV transport and a biochemical context that modulates their function [53]. Conversely, EVs contribute signaling molecules that reinforce or modify the composition and activity of SP constituents. This reciprocal interaction forms a regulatory axis crucial for sperm protection and fertilization competence [56].

One critical dimension of this crosstalk is the integration of OS and redox signaling. SP harbors enzymatic antioxidants such as SOD, CAT, and glutathione peroxidase, as well as non-enzymatic counterparts, including vitamins C, E, and glutathione [10]. EVs complement these defense systems by carrying antioxidant enzymes and stress-response proteins, delivering them directly to spermatozoa [53]. This dual-layer protection minimizes ROS-induced DNA fragmentation, lipid peroxidation, and protein oxidation, thereby safeguarding sperm genomic integrity. Dysregulation of this SP-EV oxidative balance, whether due to systemic metabolic stress, infections, or varicocele, promotes oxidative overload and male infertility [33].

The endocrine and paracrine interplay between SP and EVs further enhances their collective role. Hormones such as testosterone, estradiol, cortisol, leptin, and insulin present in SP influence sperm physiology, while EVs amplify these signals by transferring hormone receptors, signaling intermediates, and regulatory RNAs to sperm cells [57]. For instance, EV-mediated delivery of insulin receptor substrates or leptin-modulating miRNAs can modify sperm metabolic activity, linking systemic endocrine health with gamete function [58,59]. This SP-EV interaction, therefore, serves as a critical interface where systemic disorders such as obesity, diabetes, or hypogonadism manifest in reproductive dysfunction.

The immune components of SP have already been discussed and are vital for maintaining sperm viability within the female reproductive tract, where they act as protective mediators against hostile immune and oxidative challenges. SP contains immunosuppressive cytokines and prostaglandins that prevent sperm rejection in the female reproductive tract [60]. EVs augment this function by transporting immunomodulatory proteins and RNAs that condition spermatozoa and modulate local immune responses [61]. Together, these systems ensure sperm survival in the immunologically challenging environment of the female genital tract. The pathological disruption of SP-EV interactions has profound implications. Reduced EV secretion or altered cargo composition in inflammatory states results in compromised SP antioxidant and immunosuppressive capacity, leading to heightened oxidative damage and immune-mediated sperm clearance [62]. Clinical studies demonstrate that infertile men often display altered SP proteomic and EV RNA profiles, underscoring their interdependence as diagnostic reservoirs [63].

**Figure 1:**
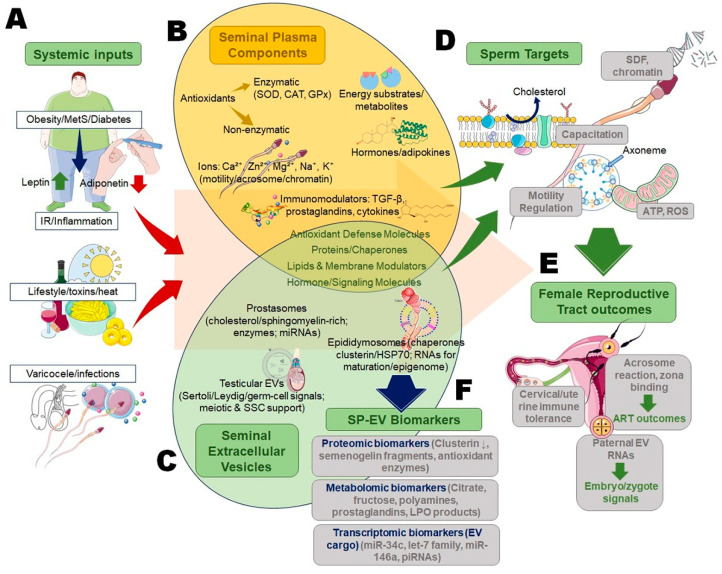
Mechanistic overview of the seminal plasma-extracellular vesicles axis in male infertility. (**A**) Systemic inputs, such as obesity, diabetes, metabolic syndrome, lifestyle toxins, heat, varicocele, and infections, disrupt oxidative balance and endocrine homeostasis, altering the SP composition and EV cargo. (**B**) SP provides enzymatic and non-enzymatic antioxidants, metabolites, ions, hormones, adipokines, and immunomodulators that regulate sperm motility, capacitation, and immune tolerance. (**C**) EV subtypes, prostasomes, epididymosomes, and testicular EVs, deliver proteins, lipids, antioxidants, receptors, and RNAs to sperm, complementing SP in redox and signaling regulation. (**D**) This integrated SP-EV antioxidant network preserves sperm DNA, membrane integrity, and motility under oxidative challenges, while its dysregulation contributes to oxidative infertility. (**E**) Within the female tract, SP and EV mediators promote immune tolerance, the acrosome reaction, and zona binding, and may also influence embryo or zygote signals. (**F**) Multi-omics profiling of SP-EV cargo has identified proteomic, metabolomic, and transcriptomic biomarkers with diagnostic, prognostic, and therapeutic utility in male infertility and ART outcomes.

From a translational perspective, the SP-EV axis is an attractive target for biomarker discovery and therapeutic modulation. A mechanistic overview of the SP-EV axis is given in Figure 1. Multi-omics profiling of SP and EV cargo provides complementary insights into sperm function and systemic health, offering greater diagnostic precision than either component alone. Moreover, engineered EVs or antioxidant supplementation strategies could be designed to restore SP-EV homeostasis in pathological infertility. Thus, EVs and SP form a synergistic molecular network that integrates oxidative, endocrine, and immunological signals in semen. Their crosstalk ensures sperm protection and fertilization competence, while their disruption underlies many unexplained cases of male infertility. Deciphering this interplay is therefore central to advancing biomarker discoveries and therapeutic strategies.

## 4. Oxidative Stress and Redox Signaling in the Seminal Plasma–Extracellular Vesicles Axis

### 4.1. Sources of Reactive Oxygen Species in Semen

ROS are inevitable byproducts of cellular metabolism, yet their overproduction within the male reproductive tract constitutes a central mechanism underlying OS-induced infertility [64]. In semen, ROS arise from multiple intrinsic and extrinsic sources, each contributing to the delicate balance between physiological signaling and pathological damage. One major endogenous source is immature spermatozoa [65]. During spermiogenesis, cytoplasmic extrusion is often incomplete, leaving behind residual cytoplasmic droplets rich in glucose-6-phosphate dehydrogenase. This enzyme enhances NADPH production, fueling NADPH oxidase activity and excessive superoxide anion generation. Immature spermatozoa are therefore disproportionately responsible for basal ROS production compared with mature sperm cells [66]. Another significant contributor is leukocytes, particularly neutrophils and macrophages, which infiltrate the male genital tract during infection, inflammation, or subclinical prostatitis [67]. Activated leukocytes generate high levels of superoxide and hydrogen peroxide during the respiratory burst. Even small leukocyte concentrations can elevate seminal ROS levels beyond physiological thresholds, overwhelming antioxidant defenses and damaging sperm DNA, proteins, and lipids [68,69]. Mitochondria within spermatozoa themselves are also central to ROS generation. Defective oxidative phosphorylation in sperm mitochondria, often triggered by ageing, varicocele, environmental toxins, or metabolic disorders, leads to electron leakage at complexes I and III of the electron transport chain. This results in the production of superoxide radicals that, if unchecked, contribute to mitochondrial dysfunction, ATP depletion, and impaired motility [70].

Exogenous factors exacerbate seminal ROS production. Cigarette smoking, alcohol consumption, heat stress, and exposure to environmental pollutants increase systemic OS, reflected within semen [71,72]. Moreover, metabolic disorders such as diabetes and obesity amplify ROS generation through systemic inflammation, insulin resistance, and dyslipidemia, further disrupting reproductive redox balance [73].

Importantly, low levels of ROS are not inherently detrimental; they are essential for sperm capacitation, hyperactivation, and acrosome reaction by activating intracellular signaling cascades [24]. The pathological state emerges when ROS production exceeds the neutralizing capacity of seminal antioxidants [10]. This threshold is commonly breached in idiopathic infertility and clinical conditions such as varicocele, infections, and metabolic syndrome [28,74]. Thus, the sources of ROS in semen represent a confluence of intrinsic sperm-derived pathways, infiltrating leukocytes, mitochondrial dysfunction, and lifestyle-related or systemic influences [70,71]. Recognizing the diversity of ROS origins highlights the need for tailored therapeutic strategies. Targeted antioxidant therapy, leukocyte suppression, and metabolic control may collectively mitigate OS and restore male fertility potential.

### 4.2. Molecular Targets of Oxidative Stress

Excessive ROS in semen inflicts damage on critical molecular targets, undermining sperm function and compromising fertilization potential [75]. The three principal targets include DNA, lipids, and proteins, each contributing uniquely to the pathophysiology of male infertility [65]. DNA fragmentation represents a hallmark outcome of OS. Hydroxyl radicals and peroxynitrite directly attack the sperm genome, inducing single- and double-strand breaks, base modifications, and chromatin cross-linking [76]. Given the limited DNA repair capacity of mature spermatozoa, oxidative lesions accumulate, leading to increased DNA fragmentation index (DFI) [77]. High DFI correlates strongly with reduced fertilization rates, impaired embryo development, and increased miscarriage risk in ART [78]. Oxidative DNA damage also generates mutagenic adducts such as 8-hydroxy-2′-deoxyguanosine (8-OHdG), which serve as molecular biomarkers of OS-induced infertility [79].

LPO is another major consequence, as sperm plasma membranes are enriched with polyunsaturated fatty acids (PUFAs). ROS attack PUFAs, generating lipid peroxides and reactive aldehydes such as malondialdehyde (MDA) and 4-hydroxynonenal (4-HNE) [64]. These byproducts destabilize the sperm membrane, impairing fluidity and ion channel function. The resulting rigidity hampers motility, capacitation, and acrosome responsiveness, directly compromising fertilization competence [65]. Protein oxidation further exacerbates functional decline. Oxidative modification of mitochondrial proteins reduces ATP synthesis, impairing sperm motility [80]. ROS-induced carbonylation of flagellar proteins compromises axonemal structure and function, while oxidation of antioxidant enzymes reduces their efficacy, amplifying redox imbalance [81]. Importantly, OS alters the function of sperm surface proteins involved in zona pellucida recognition and sperm-oocyte fusion, reducing fertilization success.

Beyond these primary targets, OS disrupts epigenetic integrity. ROS modify sperm histones and protamines, altering chromatin compaction and potentially influencing embryonic gene expression [82]. Such oxidative epimutations may mediate the paternal transmission of disease risk to offspring, underscoring transgenerational consequences [12]. Collectively, OS compromises the genomic, structural, and functional integrity of spermatozoa. The convergence of DNA fragmentation, lipid peroxidation, and protein oxidation provides a mechanistic basis for unexplained infertility, recurrent ART failure, and adverse reproductive outcomes [65,79]. Therefore, identifying molecular signatures of oxidative damage is critical for diagnosis, prognostication, and therapeutic targeting in male infertility. Table 1 summarizes the principal oxidative and endocrine alterations observed in seminal plasma and extracellular vesicles, their effects on sperm physiology, and their clinical implications in male infertility.

### 4.3. Protective Mechanisms Within Seminal Plasma and Extracellular Vesicles

Although sperm are vulnerable to OS, they retain strong defense mechanisms within SP and EVs [5]. These protective systems maintain a delicate redox equilibrium necessary for sperm function. SP harbours a diverse array of enzymatic antioxidants, including SOD, CAT, and GPx [98]. SOD catalyzes the dismutation of superoxide radicals into hydrogen peroxide, which is subsequently neutralized by CAT and GPx, preventing the accumulation of highly reactive hydroxyl radicals. In parallel, non-enzymatic antioxidants such as vitamin C, vitamin E, uric acid, taurine, and glutathione contribute to neutralizing ROS and preserving membrane integrity [10]. EVs provide an additional layer of protection by packaging and delivering antioxidant molecules directly to spermatozoa [83]. Prostasomes and epididymosomes carry enzymes such as peroxiredoxins, GPx, and thioredoxin, along with stress-response proteins like HSP70 [46]. These vesicle-bound antioxidants localize precisely to sperm membranes and mitochondria, regions particularly susceptible to oxidative insult. By delivering targeted antioxidant cargo, EVs not only prevent oxidative damage but also enhance sperm survival within the oxidative environments of the male and female reproductive tracts [5].

Beyond direct neutralization, SP and EVs also regulate redox signaling pathways. Controlled ROS levels act as signaling mediators for capacitation and hyperactivation. Antioxidants, therefore, do not indiscriminately eliminate ROS but fine-tune their concentrations, ensuring physiological signaling while preventing pathological excess. This regulation underscores the evolutionary adaptation of SP-EV systems in balancing fertility-related redox dynamics [5].

Clinical conditions such as varicocele, infections, and metabolic syndrome overwhelm SP antioxidant reserves, leading to oxidative imbalance [80]. Reduced EV secretion or impaired antioxidant loading further compromises defense mechanisms, exacerbating sperm vulnerability. Indeed, seminal total antioxidant capacity (TAC) and EV antioxidant cargo are consistently lower in infertile men compared with fertile counterparts [61]. These findings position SP and EV antioxidants as crucial guardians of sperm function. Furthermore, their measurable levels serve as potential diagnostic biomarkers of oxidative infertility. From a therapeutic perspective, strategies aimed at enhancing EV antioxidant content or supplementing exogenous antioxidants may provide effective interventions to restore redox balance and improve fertility outcomes.

### 4.4. Dysregulation and Infertility

When the balance between ROS generation and antioxidant defenses is disrupted, OS becomes pathological, leading to sperm dysfunction and infertility. This dysregulation manifests in several clinical contexts, including idiopathic male infertility, varicocele, infections, and systemic metabolic disorders [99]. In idiopathic infertility, where conventional semen parameters often appear normal, OS emerges as a hidden pathology. Elevated ROS levels and increased DNA fragmentation are frequently reported in such cases, suggesting that oxidative imbalance underlies many unexplained reproductive failures [64]. Similarly, varicocele, characterized by impaired testicular venous drainage, enhances scrotal temperature and hypoxia, thereby amplifying mitochondrial ROS production and leukocyte infiltration. This localized OS compromises spermatogenesis, sperm motility, and chromatin integrity [54]. Genital tract infections further exacerbate OS by recruiting leukocytes that release high levels of ROS during immune responses. While intended as antimicrobial agents, these ROS indiscriminately damage spermatozoa, worsening infertility. Chronic prostatitis and epididymitis exemplify conditions where persistent leukocyte activity overwhelms seminal antioxidants [31,74].

Systemic metabolic disorders, particularly obesity, diabetes, and insulin resistance, also disrupt redox balance [28,100]. Hyperglycemia and dyslipidemia increase systemic oxidative load, which translates to altered SP composition and impaired EV antioxidant cargo [92]. Endocrine crosstalk links metabolic stress to reproductive dysfunction: elevated leptin and insulin resistance correlate with increased ROS levels and reduced sperm function [101]. This integration of metabolic and reproductive pathophysiology highlights the SP-EV axis as a mediator of systemic influences on fertility. Clinically, oxidative dysregulation not only reduces natural conception rates but also impacts ART outcomes [93]. Elevated seminal ROS and DFI predict poor fertilization, impaired embryo development, and recurrent implantation failure [102]. Therefore, assessing oxidative biomarkers (e.g., 8-OHdG, MDA, TAC) has been proposed as an adjunct diagnostic tool in infertility clinics.

Therapeutically, antioxidant supplementation, varicocelectomy, infection control, and lifestyle modifications have demonstrated efficacy in restoring redox balance [84]. Emerging approaches include engineering EVs to deliver antioxidant cargo or modulating EV secretion to enhance sperm resilience. However, challenges remain in standardizing diagnostic cutoffs and tailoring therapy to patient-specific oxidative profiles [103]. Thus, dysregulation of oxidative homeostasis represents a central pathway linking diverse etiologies to male infertility. The SP-EV axis functions as both mediator and biomarker of this imbalance, underscoring its significance in advancing precision diagnostics and targeted interventions.

## 5. Endocrine Crosstalk and the Metabolic–Reproductive Axis

### 5.1. Hormones and Metabolic Regulators in Seminal Plasma

SP is a complex endocrine fluid enriched with hormones and metabolic regulators that exert profound effects on male reproductive competence. Among the most studied components are sex hormones, including testosterone, estradiol, and dihydrotestosterone, which are detectable in measurable concentrations within SP [104]. These androgens, secreted by the testes and prostate, are indispensable for spermatogenesis, sperm motility, and capacitation. Estradiol, though traditionally associated with female reproduction, also plays a vital role in male fertility by modulating acrosome responsiveness and maintaining sperm genomic stability [104].

Beyond sex steroids, SP harbours peptide hormones and metabolic regulators such as leptin, insulin, adiponectin, ghrelin, cortisol, and inhibin B [85]. These molecules reflect both systemic endocrine status and local glandular contributions. Leptin, primarily secreted by adipose tissue, is present in SP and correlates with sperm motility and morphology, though elevated levels in obese men are often associated with impaired sperm function [27]. Insulin, similarly, not only regulates glucose homeostasis but also modulates sperm metabolism and capacitation [86]. Adiponectin, an insulin-sensitizing adipokine, is implicated in maintaining anti-inflammatory and antioxidative conditions within the ejaculate [87,105]. Cortisol, a glucocorticoid stress hormone, exerts immunomodulatory effects but can impair fertility when chronically elevated [106]. Inhibin B, produced by Sertoli cells, is another critical SP marker, reflecting seminiferous tubule function and spermatogenic activity. Reduced levels are consistently observed in men with impaired spermatogenesis, making it a potential diagnostic indicator [107]. Collectively, these hormonal mediators integrate systemic metabolic health with local reproductive functions. Thus, the concentration and balance of these hormones are influenced by systemic disorders. Obesity, metabolic syndrome, and type 2 diabetes alter leptin, insulin, and adiponectin profiles, leading to dysregulated sperm metabolism and heightened OS. PCOS in female partners further complicates the endocrine milieu, as reciprocal metabolic stressors influence male reproductive hormones [9]. Thus, the hormonal and metabolic profile of SP serves as both a functional regulator of sperm physiology and a biomarker of systemic health. Its endocrine composition integrates metabolic stress, oxidative balance, and reproductive capacity, thereby positioning SP as a unique window into the metabolic-reproductive axis in male infertility.

### 5.2. Extracellular Vesicles-Mediated Hormonal and Signaling Interactions

EVs in semen provide an additional dimension to endocrine communication by functioning as carriers of hormone receptors, signaling intermediates, and regulatory RNAs [5]. Unlike soluble factors in SP, EVs deliver their cargo in a protected vesicular form, ensuring stability and targeted transfer to spermatozoa and surrounding cells. This EV-mediated signaling establishes a paracrine and autocrine regulatory network central to male fertility. One of the most striking features of EVs is their ability to transfer hormone receptors [36]. For example, prostasomes have been shown to deliver androgen, estrogen, and insulin receptor fragments to sperm, thereby modulating their sensitivity to systemic endocrine cues. This vesicle-mediated receptor transfer can enhance sperm responsiveness in environments where free hormone concentrations may be suboptimal [36,88]. Similarly, epididymosomes carry G-protein-coupled receptor components that participate in capacitation-related signaling [39]. These receptors are believed to influence steroid-mediated signaling pathways that regulate capacitation, acrosome reaction, and sperm metabolic activity, thereby integrating endocrine and metabolic control at the gamete level. The functional relevance of these receptor transfers is increasingly recognized. Androgen receptor signaling modulates sperm plasma membrane remodeling and cholesterol efflux, thereby influencing capacitation readiness. Estrogen receptors play a role in regulating calcium channels and contribute to the timing of the acrosome reaction. In parallel, insulin receptor fragments influence glucose uptake and mitochondrial energy production, supporting motility and ATP homeostasis under variable metabolic conditions. Together, these vesicle-mediated receptor transfers enhance the ability of sperm to respond to local hormonal gradients within the reproductive tract.

EVs also transport signaling proteins and enzymes that directly affect sperm physiology. Kinases, phosphatases, and ion channel regulators packaged within EVs influence motility, acrosome reaction, and plasma membrane remodeling [52]. Importantly, many of these enzymes modulate redox balance, thereby linking endocrine and oxidative signaling pathways. Perhaps the most transformative role of EVs lies in their delivery of regulatory RNAs. MicroRNAs (miRNAs), small interfering RNAs (siRNAs), and piRNAs carried by EVs can reprogram sperm transcriptomes and post-fertilization gene expression [108]. For instance, EV-derived miRNAs targeting insulin receptor pathways modulate sperm energy metabolism, while those regulating leptin signaling influence motility. These vesicle-borne RNAs also mediate paternal epigenetic inheritance, potentially linking systemic metabolic conditions with transgenerational reproductive outcomes [45].

Critically, alterations in EV-mediated endocrine signaling are associated with infertility. Infertile men frequently exhibit altered EV RNA profiles, reduced receptor cargo, and impaired vesicle-sperm fusion capacity. These changes translate into diminished hormonal responsiveness, impaired capacitation, and reduced ART success [45]. Moreover, systemic metabolic disorders such as obesity and diabetes alter the composition of semen EVs, thereby disturbing endocrine-metabolic integration at the gamete level [5]. Thus, semen EVs represent a sophisticated communication system that integrates hormonal, metabolic, and redox signaling. Their ability to deliver protected, functional biomolecules distinguishes them from soluble SP hormones, and their dysregulation directly links systemic health with sperm competence. From a translational standpoint, EV-mediated hormonal signaling represents a promising frontier for biomarker discovery and therapeutic intervention in male infertility.

### 5.3. Integration with Systemic Metabolic Disorders

The SP-EV axis does not operate in isolation but is profoundly influenced by systemic metabolic disorders such as obesity, diabetes, insulin resistance, and metabolic syndrome. These conditions not only alter systemic hormone levels but also reshape the molecular composition of SP and EVs, leading to impaired sperm function and infertility [45].

Obesity is one of the most prominent disruptors. Adipose tissue dysfunction leads to elevated leptin levels, reduced adiponectin levels, and chronic low-grade inflammation. In SP, this manifests as altered concentrations of leptin and proinflammatory cytokines, which impair sperm motility, increase OS, and reduce capacitation potential [109]. EVs derived from obese men exhibit altered miRNA and protein cargo, including signals that dysregulate mitochondrial metabolism and enhance ROS generation [43]. Diabetes mellitus introduces additional layers of dysregulation. Hyperglycemia and insulin resistance increase advanced glycation end-products (AGEs), systemic OS, and inflammation [86]. In SP, this translates into reduced antioxidant capacity, increased oxidative damage, and altered hormonal profiles [94]. EVs from diabetic patients frequently carry miRNAs targeting insulin signaling and mitochondrial pathways, resulting in impaired sperm energy metabolism and reduced fertilization potential [110]. Metabolic syndrome, characterized by central obesity, dyslipidemia, hypertension, and insulin resistance, compounds these effects by creating a pro-inflammatory and pro-oxidative systemic environment [100]. SP composition in men with metabolic syndrome reflects this imbalance, with decreased TAC, altered proteomic signatures, and hormone dysregulation. EV cargo mirrors these changes, carrying stress-response proteins and dysregulated miRNAs that impair sperm function [5]. Interestingly, the integration of systemic disorders with the reproductive microenvironment is a bidirectional process. While systemic metabolic stress alters SP and EV composition, dysfunction within the SP-EV axis can exacerbate systemic oxidative load and contribute to broader endocrine disturbances. This interdependence underscores the SP-EV axis as a critical mediator of the metabolic-reproductive interface [5].

Clinically, infertile men with metabolic disorders exhibit reduced sperm quality, increased DNA fragmentation, and lower ART success [102]. Importantly, profiling SP hormones and EV cargo offers diagnostic potential to stratify infertility risk in men with systemic metabolic disease. Therapeutically, lifestyle modification, weight reduction, and metabolic control improve SP antioxidant capacity and normalize EV composition, underscoring the reversibility of these molecular disruptions [71]. Thus, systemic metabolic disorders profoundly influence male fertility through alterations in the SP-EV molecular network. Understanding this integration not only explains the high prevalence of infertility in obese and diabetic men but also highlights novel avenues for biomarker discovery and targeted interventions that bridge metabolic health and reproductive success.

## 6. Seminal Plasma and Extracellular Vesicles as Biomarker Reservoirs

### 6.1. Proteomic, Metabolomic and Transcriptomic Approaches

Advances in high-throughput omics technologies have transformed our understanding of SP and EVs as molecular reservoirs [95]. Table 2 summarizes the major categories of seminal plasma and extracellular vesicle proteins, metabolites, and signaling molecules associated with oxidative stress and infertility, emphasizing representative functional groups. Proteomic, metabolomic, and transcriptomic profiling provide comprehensive maps of biomolecules that not only reflect sperm function but also systemic influences on male reproductive health. These multi-omics approaches are particularly valuable in deciphering complex, multifactorial causes of infertility where traditional semen analysis falls short [96]. Increasingly, integrative approaches combining proteomic data with transcriptomic and functional readouts have shown improved diagnostic resolution compared with single-marker analyses, reinforcing the value of multi-parameter biomarker signatures in male fertility assessment.

Proteomics has identified thousands of proteins within SP and EVs, including enzymes, structural proteins, cytokines, growth factors, and antioxidants [97]. Mass spectrometry-based studies have identified key candidates, including clusterin, semenogelin, lactoferrin, and prostatic acid phosphatase, many of which are involved in sperm motility, capacitation, and immune modulation [111]. In EVs, proteins like tetraspanins (CD9, CD63), annexins, and HSPs act as biomarkers of vesicle origin and function. Importantly, proteomic alterations are consistently observed in infertile men, with reduced antioxidant enzymes and dysregulated immune proteins being recurrent features [112]. Within the proteomic landscape, the glycoproteomic features of seminal plasma and extracellular vesicles are particularly distinctive. Protein glycosylation modulates molecular stability, immune recognition, and sperm-tract interactions, thereby contributing to sperm maturation and protection. Altered glycosylation patterns may disrupt these processes, linking glycoprotein composition with male fertility potential [113].

Metabolomics complement proteomics by profiling small molecules that mediate energy metabolism, redox status, and signaling. Nuclear magnetic resonance (NMR) and liquid chromatography-mass spectrometry (LC-MS) approaches have uncovered metabolites such as citrate, fructose, polyamines, and prostaglandins as critical regulators of sperm motility and fertilization capacity [114]. Altered metabolic signatures, including elevated lipid peroxidation products and reduced antioxidant metabolites, are characteristic of OS-related infertility. Metabolomic profiling of SP and EVs, therefore, provides a dynamic snapshot of the metabolic-reproductive axis [115].

Transcriptomics, particularly small RNA sequencing, has revealed that semen EVs carry a diverse cargo of mRNAs, miRNAs, and piRNAs [116]. These transcripts influence post-transcriptional regulation of sperm function and contribute to paternal epigenetic inheritance. For instance, differential expression of miRNAs regulating insulin signaling, mitochondrial function, and apoptosis has been linked to idiopathic infertility [89]. piRNAs within EVs are increasingly recognized as regulators of genome stability in sperm and early embryos [90].

The integration of proteomic, metabolomic, and transcriptomic datasets provides a powerful systems biology approach to male infertility [96]. However, challenges remain, including variability in sample handling, methodological inconsistencies, and the need for standardized EV isolation protocols. Multi-omics integration, when combined with machine learning, offers promise in identifying robust biomarker signatures [96]. Ultimately, these approaches move beyond descriptive analysis to predictive and mechanistic insights, establishing SP and EVs as rich molecular repositories that bridge basic research and clinical application in male infertility.

**Table 2:** Seminal plasma and extracellular vesicles biomarkers: Translational potential in male infertility.

Biomarker Type	Examples (SP/EV)	Clinical Context	Diagnostic Utility	Prognostic/Therapeutic Potential	References
**Proteins**	Clusterin, semenogelin fragments, heat shock proteins (HSP70, HSP90), antioxidant enzymes (GPx, peroxiredoxins), annexins, tetraspanins (CD9, CD63), lactoferrin, prostatic acid phosphatase	Idiopathic infertility, oxidative infertility	Distinguish fertile vs. infertile men	Predict sperm resilience; guide antioxidant therapy	[36,47,63,83,112,113]
**Metabolites**	Citrate, fructose, polyamines, prostaglandins, lipid peroxidation products (MDA, 4-HNE)	Metabolic-associated infertility, varicocele	Reflect sperm energy metabolism & redox state	Monitor lifestyle/therapeutic interventions	[65,115,116]
**Hormones & adipokines**	Testosterone, estradiol, cortisol, leptin, insulin, adiponectin	Obesity, diabetes, endocrine-linked infertility	Stratify patients with metabolic comorbidities	Informed ART treatment selection	[26,28,85,87,88,105,106,107]
**RNAs (EV cargo)**	miR-34c, miR-146a, let-7 family, piRNAs	Idiopathic infertility, ART failure	High sensitivity/specificity for male infertility subtypes	Predict embryo quality and implantation success	[44,50,89,90,91,117]
**Composite panels**	Multi-omics SP + EV signatures	All major infertility categories	Improved diagnostic accuracy vs. semen analysis	Enable precision medicine, targeted therapy	[96,97]

### 6.2. Diagnostic Potential in Male Infertility

SP and EVs represent highly accessible, non-invasive reservoirs for biomarker discovery, offering diagnostic potential that exceeds that of conventional semen analysis. Traditional parameters such as sperm concentration, motility, and morphology provide limited predictive power, especially in idiopathic infertility, where up to 30% of cases remain unexplained [91]. Molecular profiling of SP and EVs provides a richer diagnostic landscape, capturing underlying oxidative, endocrine, and metabolic disruptions. One major advantage of SP and EV biomarkers lies in their non-invasive sampling [5]. Unlike testicular biopsies, ejaculate collection is simple, repeatable, and acceptable to patients, enabling longitudinal monitoring. Moreover, SP and EVs directly reflect the reproductive microenvironment, providing localized insights not captured by serum markers.

Specific biomarker candidates have already demonstrated diagnostic utility. In SP, elevated levels of OS markers such as 8-OHdG and MDA correlate strongly with sperm DNA fragmentation and infertility risk [76]. Reduced levels of antioxidant enzymes (SOD, CAT, GPx) further stratify oxidative infertility [10]. Proteomic studies identified decreased clusterin and altered semenogelin fragments as consistent features of infertile men [111]. Similarly, EV cargo provides unique diagnostic opportunities: differential miRNA expression patterns (e.g., miR-34c, miR-146a, miR-19b) distinguish fertile from infertile men with high sensitivity and specificity [43].

Biomarker signatures can also differentiate infertility subtypes. In varicocele, SP exhibits altered proteomic and metabolomic profiles indicative of hypoxia and OS, while EVs carry stress-response proteins and miRNAs [54]. In idiopathic infertility, EV RNA signatures often reveal disruptions in endocrine and mitochondrial pathways not apparent in semen analysis. In metabolic-associated infertility, altered leptin, insulin, and adiponectin levels in SP, combined with EV miRNAs targeting insulin resistance pathways, provide diagnostic specificity. Significantly, biomarker assays are moving toward clinical translation. Assays for TAC, lipid peroxidation, and sperm DNA fragmentation are already incorporated in some clinics. Expanding to SP-EV multi-marker panels could substantially improve diagnostic accuracy, enabling personalized treatment strategies [63]. However, challenges remain in assay standardization, cost, and inter-individual variability. Thus, SP and EV molecular biomarkers hold significant promise as non-invasive diagnostic tools. Their ability to reflect OS, endocrine dysregulation, and metabolic status offers a precision medicine approach to male infertility diagnosis, bridging the gap between molecular pathophysiology and clinical application.

### 6.3. Prognostic and Therapeutic Implications

Beyond diagnosis, SP and EVs have significant potential as prognostic markers and therapeutic targets in male infertility. The dynamic composition of SP and EV cargo reflects ongoing reproductive and systemic processes, offering predictive insights into ART outcomes and guiding therapeutic interventions [5]. Prognostic value has been demonstrated in multiple studies. High levels of sperm DNA fragmentation, reflected by oxidative biomarkers in SP, correlate with reduced fertilization rates, impaired blastocyst development, and increased miscarriage rates in ART cycles [102]. Similarly, EV miRNA profiles can predict intracytoplasmic sperm injection (ICSI) success. For example, altered expression of sperm-specific miRNAs such as miR-34c and let-7 family members has been associated with poor embryo quality and implantation failure [118]. SP proteomic signatures, including reduced clusterin and altered prostasome proteins, also serve as prognostic indicators of ART success. Therapeutically, SP and EVs represent promising avenues for intervention. Antioxidant supplementation, while widely used, often yields inconsistent results due to a lack of patient stratification. Profiling SP antioxidant capacity and EV cargo could identify subgroups most likely to benefit, thereby personalizing therapy. Similarly, lifestyle modifications that improve metabolic health, weight reduction, improve insulin sensitivity, and reduce systemic inflammation are reflected in improved SP-EV profiles, supporting fertility restoration [71]. Innovative therapeutic approaches are emerging from the field of EV biology itself. EVs can be harnessed as therapeutic vehicles, engineered to deliver antioxidant enzymes, signaling proteins, or regulatory RNAs directly to spermatozoa. Preclinical studies suggest that EV-based interventions could restore capacitation, reduce DNA fragmentation, and enhance fertilization outcomes. Moreover, modulating EV biogenesis and cargo loading may offer a means of correcting SP-EV dysregulation in metabolic or oxidative infertility.

From a prognostic perspective, SP and EV profiling may also aid in the selection of treatment. For example, couples with high sperm DNA fragmentation may benefit from testicular sperm extraction for ICSI rather than ejaculated sperm [102]. Similarly, biomarker-informed antioxidant therapy could improve ART outcomes by targeting redox imbalance [93]. Despite these advances, clinical translation requires addressing challenges such as assay standardization, reproducibility, and regulatory approval for EV-based therapies. Integration of multi-omics data with machine learning could generate predictive algorithms that stratify patients for prognosis and therapy more effectively [95,96]. Thus, SP and EVs hold dual promise as prognostic indicators of ART success and as therapeutic targets for restoring male fertility. Their integration into clinical workflows could shift infertility management from empirical approaches to precision medicine, offering tailored interventions that improve reproductive outcomes.

## 7. Translational Perspectives and Future Directions

The recognition of SP and EVs as dynamic regulators of sperm function has significant translational implications for the diagnosis, prognosis, and treatment of male infertility. However, despite substantial advances in molecular profiling, the clinical application of SP-EV research remains in its infancy. Moving toward translation requires addressing methodological, biological, and clinical challenges while integrating emerging technologies to harness the full potential of these biomolecular reservoirs. One of the foremost limitations lies in the standardization of EV isolation and characterization. Currently, diverse methods, ultracentrifugation, size-exclusion chromatography, precipitation, and immunoaffinity capture, yield EV populations of variable purity and reproducibility [44]. This heterogeneity undermines biomarker validation and complicates cross-study comparisons. Establishing consensus guidelines for EV isolation, characterization, and reporting, akin to the Minimal Information for Studies of Extracellular Vesicles (MISEV) framework, will be essential to enhance reproducibility and clinical applicability [117]. Similarly, inter-individual variability in SP composition, influenced by factors such as age, lifestyle, diet, and systemic metabolic disorders, presents challenges to biomarker consistency. Large-scale, multicentric studies that incorporate diverse patient cohorts are necessary to distinguish pathological signatures from physiological variability. Ethical considerations are also critical for the clinical translation of EV-based biomarkers and therapies in ART. These include ensuring the safety and standardization of EV isolation and modification, minimizing unintended biological effects, and addressing issues of informed consent, data privacy, and equitable access to advanced reproductive diagnostics. Careful ethical oversight and regulatory frameworks are therefore essential for responsible clinical implementation.

The integration of multi-omics approaches represents a promising future direction. By combining proteomics, metabolomics, and transcriptomics, a more comprehensive and robust molecular fingerprint of SP and EVs can be generated [95,96]. Machine learning and artificial intelligence platforms further augment this potential by identifying predictive biomarker panels with high sensitivity and specificity. Such integrative models could stratify patients into distinct infertility subtypes, enabling personalized treatment plans [119]. From a therapeutic standpoint, EV-based interventions offer exciting opportunities. EVs can be engineered as natural nanocarriers for targeted delivery of antioxidants, signaling proteins, or small RNAs to spermatozoa, potentially correcting oxidative imbalance or endocrine dysfunction. Likewise, modulation of EV biogenesis or cargo loading may provide novel strategies to enhance sperm resilience in adverse reproductive environments. While preclinical studies are encouraging, rigorous clinical trials are required to establish the safety, efficacy, and scalability of EV-based therapies. The concept of personalized medicine is particularly relevant in male infertility—a field historically managed with empirical treatments. Profiling SP and EV signatures could identify patients who would benefit most from antioxidant supplementation, lifestyle modifications, or specific ART strategies such as testicular sperm extraction. Furthermore, predictive SP-EV biomarkers could guide ART outcome expectations, reducing emotional and financial burdens on couples undergoing treatment.

Another future perspective is the exploration of transgenerational implications. Given that EVs carry regulatory RNAs influencing early embryonic development, alterations in SP-EV cargo may mediate paternal transmission of metabolic or epigenetic risk factors to offspring [120]. Longitudinal studies examining the impact of paternal SP-EV signatures on pregnancy outcomes and child health are urgently needed to expand the relevance of these findings beyond infertility to broader reproductive and developmental health. Thus, the SP-EV axis represents a molecular gatekeeper of male fertility, offering unparalleled opportunities for biomarker discovery and therapeutic innovation. Bridging bench research with bedside applications requires methodological standardization, multi-omics integration, and well-designed clinical studies. By advancing these translational directions, SP and EVs may not only revolutionize infertility diagnosis and treatment but also provide critical insights into the systemic integration of metabolism, reproduction, and long-term health outcomes.

## 8. Conclusions

SP and EVs should be viewed as components of a single, integrated regulatory system rather than as independent contributors to sperm biology. Acting together, they form a highly coordinated molecular network that supports sperm survival, maturation, and fertilization competence through the regulation of redox balance, hormonal responsiveness, immune modulation, and intracellular signaling. This unified perspective provides a clearer understanding of how normal physiological variation is maintained and how pathological alterations arise in male infertility.

A major insight from this review is the central role of OS in disrupting sperm function. Excessive ROS-mediated damage to DNA, lipids, and proteins undermines sperm integrity and reproductive outcomes, particularly in conditions such as varicocele, metabolic disorders, infection, ageing, and idiopathic infertility. Although SP and EVs provide layered antioxidant protection, these defenses are often insufficient under pathological conditions, leading to sustained oxidative imbalance and functional decline.

The endocrine and metabolic dimensions of the SP–EV axis further emphasize the close connection between systemic health and male reproductive capacity. Hormones and metabolic regulators present in SP, together with EV-mediated transfer of receptors and regulatory RNAs, create a molecular interface through which obesity, diabetes, and related metabolic disturbances directly influence sperm function. Recognizing this interaction reinforces the concept of male infertility as a condition shaped by both local reproductive and broader systemic factors.

Beyond advancing mechanistic understanding, SP and EVs represent valuable reservoirs for clinically relevant biomarkers. Multi-omics studies have identified proteomic, metabolomic, and transcriptomic signatures that can distinguish fertile from infertile men and predict ART outcomes. These findings support the development of SP–EV-based diagnostic tools, biomarker-guided therapeutic strategies, and emerging EV-based interventions. Overall, SP and EVs are dynamic regulators whose dysregulation is linked to oxidative imbalance, endocrine disruption, and impaired fertility. Continued integrative and translational research is essential to fully realize their potential in improving the diagnosis, management, and long-term understanding of male reproductive health.

## Figures and Tables

**Table 1:** Clinical implications of oxidative stress and endocrine crosstalk in the seminal plasma-extracellular vesicles axis.

Mechanism	SP/EV Mediators	Clinical Impact on Sperm Function	Associated Clinical Conditions	Diagnostic/Prognostic Relevance	References
**OS imbalance**	↓ SOD, CAT, GPx; ↑ ROS, MDA, 8-OHdG	DNA fragmentation, lipid peroxidation, impaired motility	Varicocele, idiopathic infertility, infection, aging	Oxidative stress markers predict ART outcomes	[11,65,76,77,80,81,83,84]
**Endocrine disruption**	Altered testosterone, estradiol, inhibin B, leptin, insulin in SP; EVs carrying hormone receptors & miRNAs	Impaired capacitation, motility, energy metabolism	Obesity, diabetes, metabolic syndrome	Hormonal profiling aids patient stratification	[23,26,28,37,44,85,86,87,88,89,90,91]
**Metabolic–reproductive crosstalk**	SP adipokines (leptin, adiponectin); EV miRNAs targeting insulin signaling	Reduced sperm energy efficiency, ROS overproduction	Obesity, PCOS-linked male partners	Candidate biomarkers for metabolic-associated infertility	[28,29,30,44,60,87,92,93,94,95,96,97]
**Immune dysregulation**	Prostaglandins, cytokines in SP; EV immunomodulatory proteins	Inadequate immune tolerance, sperm clearance	Chronic prostatitis, genital tract inflammation	Potential therapeutic target for immune-modulation	[24,32,61,62,68,75]

## Data Availability

No new data were created or analyzed in this study. Data sharing is not applicable to this article.

## Abbreviations

The following abbreviations are used in this manuscript:

4-HNE	4-hydroxynonenal
8-OHdG	8-hydroxy-2′-deoxyguanosine
AGEs	advanced glycation end-products
ART	assisted reproductive technologies
cAMP	cyclic AMP
CAT	catalase
DFI	DNA fragmentation index
EVs	extracellular vesicles
GPx	glutathione peroxidase
HSP	heat shock protein
ICSI	intracytoplasmic sperm injection
LC-MS	liquid chromatography-mass spectrometry
MDA	malondialdehyde
MISEV	minimal information for studies of extracellular vesicles
miRNAs	microRNAs
NMR	nuclear magnetic resonance
OS	oxidative stress
PCOS	polycystic ovary syndrome
PUFAs	polyunsaturated fatty acids
piRNAs	piwi-interacting RNAs
ROS	reactive oxygen species
SOD	superoxide dismutase
SP	seminal plasma
siRNA	small interfering RNA
TAC	total antioxidant capacity
